# Ultrasound and Clinical Alterations in the Foot of Children with Obesity and Diabetes

**DOI:** 10.3390/diagnostics13172781

**Published:** 2023-08-28

**Authors:** Martina Pappalardo, Laura Gori, Emioli Randazzo, Riccardo Morganti, Michelangelo Scaglione, Margherita Valiani, Alessandra Beni, Maria Di Cicco, Diego G. Peroni, Ferdinando Franzoni, Pasquale Comberiati

**Affiliations:** 1 Azienda Ospedaliero Universitaria Pisana, UO Pediatria Universitaria, 56126 Pisa, Italy; 2 Section of Statistics, University Hospital of Pisa, 56126 Pisa, Italy; 3 First Orthopedic and Traumatologic Clinic, University of Pisa, 56126 Pisa, Italy; 4 Department of Clinical and Experimental Medicine, University of Pisa, 56126 Pisa, Italy

**Keywords:** anthropometry, body mass, diabetes, foot, obesity, plantar fascia, posture, ultrasound

## Abstract

Background. Alterations in plantar soft tissues are often reported in adults with diabetes, whereas data on children are conflicting. Also, the extent of foot damage caused by excess body fat in children has not been fully characterized yet. This study aimed to address the relationship between body mass and structural changes of the foot in children and adolescents with and without diabetes. Methods. In a case-control study, 43 participants (age 13 ± 2.6 years) were recruited, 29 (67%) with type 1 diabetes (T1D) and 14 (33%) controls. Anthropometric parameters [body mass index (BMI), waist circumference (WC), and waist-to-height ratio (WHtR)], foot posture index-6 (FPI-6) for static foot posture, and navicular drop test (NDT) for medial longitudinal arch height (MLA) were measured in all participants. The thickness of the midfoot plantar fascia (MPF) and medial midfoot fat pad (MMFP) were quantified using ultrasound. Results. No differences in clinical and ultrasonographical parameters were observed between the study groups. MMFP thickness was correlated with MPF thickness (*p* = 0.027). MMFP and MPF thicknesses were positively associated with BMI (*p* < 0.001 and *p* = 0.013, respectively), WC (*p* < 0.001 and *p* = 0.013), and WHtR (*p* < 0.001 and *p* = 0.026). The NDT measured on the right and left foot correlated with WHtR (*p* = 0.038 and *p* = 0.009, respectively), but not with WC and BMI. Conclusions. Children with T1D show structural alterations of plantar soft tissues which seem related to body mass increase rather than diabetes pathology. Ultrasound is a valuable tool to assess early structural changes of the foot in young people with an elevated BMI.

## 1. Introduction

Excess body fat mass is associated with musculoskeletal complications and alterations of growth patterns of the limbs and trunk in children [1]. Children with obesity show accelerated skeletal maturation, are taller, and have relatively shorter legs compared to their leaner peers [2,3]. The feet of children who are overweight or obese are characterized by structural lowering of the medial longitudinal arch (MLA), large footprints, an increase in plantar pressure in correspondence of the mid-lateral regions of the midfoot and forefoot, and an increase in the medial midfoot fat pad (MMFP) thickness [1,4,5,6].

A greater MMFP thickness is hypothesized by some authors to have a protective function for the development of the bone architecture of the MLA [1]. However, Riddiford-Harland et al. [5], did not detect a strong association between midfoot plantar pressures and excess fat padding, thus postulating that the MMFP reflects excess body fat mass rather than having a load adaptation function.

Currently, there is a paucity of data on the extent of plantar soft tissue alterations induced by excess body fat and how this can affect the structure of the plantar longitudinal arch in growing children, particularly in those with type 1 diabetes (T1D).

Patients with diabetes are prone to ulceration of the foot, also referred to as diabetic foot syndrome, due to diabetic neuropathy and peripheral artery disease. The risk factors for diabetic foot complications include obesity, worse metabolic control, hypertension, and the duration of the disease [7]. Other than neuropathic ulcers, patients with diabetes show skin changes that make them susceptible to injury from minor trauma [8]. Alterations in plantar skin and derma thickness are reported in adults with diabetes [9]. However, data on plantar soft tissue thickness in the pediatric population with diabetes are conflicting. An increase in skin thickness on the dorsum of the hands of adolescents with T1D has been reported by a previous study [10], whereas a more recent quantitative evaluation did not find any difference in the plantar skin thickness of adolescents with and without T1D [11]. However, the latter study found a thickening of plantar aponeurosis in adolescents with T1D. This alteration was significantly associated with a higher BMI but did not appear to alter the plantar arch height or pressure [11].

Early non-invasive recognition of alterations in plantar soft tissues is important in growing children with T1D. In this study, we used clinical indexes and ultrasound to assess the relationship between excess body mass and structural changes of both the plantar fat pad and plantar aponeurosis in children with T1D and control peers without diabetes.

## 2. Materials and Methods

### 2.1. Study Design and Patients

This was an observational case-control study. The study group of children and adolescents (age range 7–16 years) with T1D were recruited from the pediatric Diabetology and Endocrinology Unit of the University Hospital of Pisa, Italy between April and July 2022. The control group consisted of children and adolescents without diabetes, matched by age, age group (i.e., prepubertal, pubertal, and post-pubertal), gender, and BMI categories (i.e., underweight, normal weight, overweight, obesity) with T1D patients. Participants in both groups were considered not eligible if they had any of the following: musculoskeletal diseases, neurological diseases, infectious diseases, and pain in the lower limbs or foot at the time of the evaluations. The study was approved by the local Institutional Review Board. Informed consent was obtained from all participants or their legal guardians.

### 2.2. Anthropometry

Weight, height, BMI, Waist Circumference (WC), and Waist to Height Ratio (WHtR) were assessed in each participant. Standing height was measured using a wall pole. Body mass was measured to the nearest 0.1 kg using TANITA MC-780MA scales, which were also used to calculate the BMI. Using the pediatric BMI classification tables of the Italian Society of Pediatric Endocrinology and Diabetology (SIEDP) [12], participants were divided into the following four categories: underweight (BMI < 5th percentile), normal weight (BMI between 5th and 84th percentile), overweight (BMI between 85th and 94th percentile), and obese (BMI ≥ 95th percentile). WC was measured at navel height using a tape measure with an approximation of 0.1 cm. The anthropometric index WHtR is a measure of the body fat distribution and is calculated by dividing WC by height, both measured in the same units. A 0.5 cut-off regardless of gender was used, as previously described [13].

### 2.3. Clinical Measurements of the Foot

The Foot Posture Index-6 (FPI-6), a validated and widely applied index in podiatry clinical practice and research [14], was used to assess the static alignment of the foot on all three planes and classify the type of foot posture (normal, pronated, more pronated, supinated, or more supinated). During the evaluations, each participant was asked to stand still in a relaxed position for five minutes, then to look straight ahead and move their feet up and down on the spot to find a natural, erect position. The operator assured that the patient did not rotate the body during the evaluation and measured the six parameters for each foot (i.e., palpation of the head of the talus; supra and sub malleolar curves; inversion/eversion of the calcaneus; talonavicular congruence; the height of the medial arch; adduction/abduction of the forefoot) assigning a score that could vary from −2 to + 2 for each item. The sum of the six scores of each foot gave a final value between −12 and +12, which was compared with the reference values to determine the posture of the foot [14].

MLA was assessed using the Navicular Drop Test (NDT). NDT is a quick, easy, and inexpensive test that shows the difference (in mm) in the height of the navicular tuberosity between two positions: the neutral subtalar position and the relaxed posture [15]. High values of NDT (cut off ≥10 mm) are associated with a low MLA and pronated foot [16]. The method used for the NDT is described by Brody [15] and Zuil -Escobar [16].

### 2.4. Ultrasound Examination of the Foot

**Figure 1:**
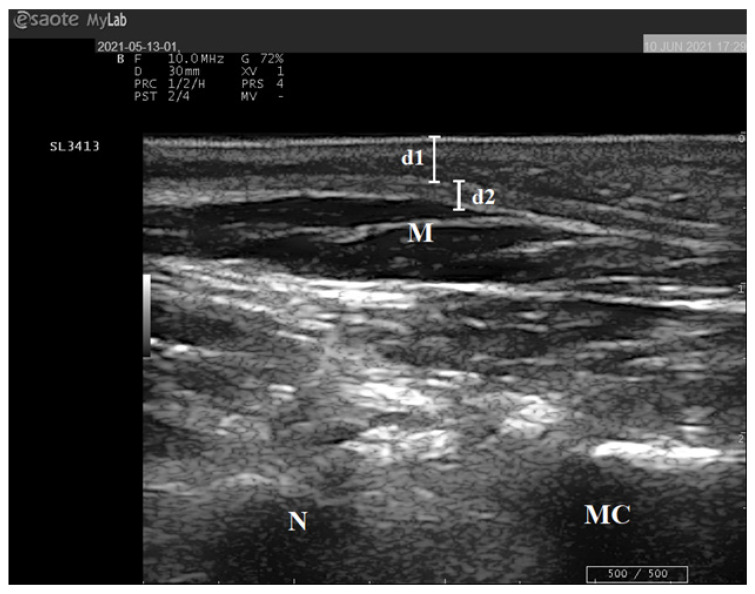
Ultrasound of the medial midfoot plantar surface in an eight-year-old healthy girl. (d1): the thickness of MMFP; (d2): the thickness of MPF; (M): short flexor muscle of the fingers; (N): navicular bone; (MC): medial cuneiform bone.

All ultrasound evaluations were performed by the same operator (L.G.) with ten years of experience in pediatric ultrasound. An ESAOTE MyLabSat portable ultrasound system with a multifrequency linear probe from 5–10 MHz with soft tissue presets (maximum depth 4 cm) was used to quantify the thickness of the MMFP and the midfoot plantar fascia (MPF) in the left and right feet of each participant (Figure 1). The method described by Riddiford-Harland DL [17] was used for all of the measurements: while the participant sat with the lower extremity flexed and the knee and foot held in a relaxed position, the operator placed the transducer on the medial midfoot plantar surface in line with the area dorso-navicular surface previously identified. The mean of the measures taken from the right and left foot was used to determine the thickness of the MMFP and the MPF of each patient.

### 2.5. Statistical Analysis

Continuous variables were summarized as mean and standard deviation (SD), while categorical variables were summarized as absolute frequency and percentage (%). To compare the differences between the two groups (diabetes vs. controls), the chi-square test was used for categorical variables, and *T*-test was used for continuous variables. One-way ANOVA was used to compare the BMI categories with MMFP and MPF thicknesses, followed by multiple comparisons with the Bonferroni method. Pearson correlation was performed to evaluate the relationship between anthropometric, clinical, and ultrasound parameters. A *p*-value of <0.05 was considered statistically significant. The statistical analysis was performed with SPSS (Statistical Package of Social Sciences, Chicago, IL, USA) v.27 techno.

## 3. Results

### 3.1. Characteristics of the Study Population

The study cohort consisted of 43 children (mean age, 13 ± 2.6 years), 23 (53.5%) of whom were males, 29 (67%) had T1D, and 14 (33%) were in the control group. Table 1 shows the demographical, clinical, and ultrasonographical characteristics of the two study groups. The mean age of the children with T1D was 12.9 ± 2.7 years, the mean duration of T1D was 5.7 ± 2.9 years, and the mean glycated hemoglobin (HbA1c) was 45.4 ± 7.07 mmol/mol IFFC (or 6.3 ± 1.4% DCCT). The children with T1D had a higher BMI than the controls, but there was no significant difference in terms of age, sex, and frequency of BMI categories between the children with T1D and the controls.

### 3.2. Relationship between Clinical Measurements of the Foot and Anthropometric Parameters

No significant differences in static foot posture, as measured with the FPI-6, and in the MLA, as measured with the NDT, were found when the TD1 children were compared with the controls (Table 1). Also, no correlation was found between HbA1c and either static food posture or MLA (data not shown). FPI-6 values showed a moderate-to-high positive correlation with NDT values (right foot r = 0.60, *p* < 0.001; left foot r = 0.73, *p* <0.001). The NDT measured on the right and left foot correlated with WHtR (r = 0.32, *p* = 0.038; and r = 0.39, *p* = 0.009, respectively), but not with WC and BMI. No correlations between FPI-6 and anthropometric variables were found (Table 2).

### 3.3. Relationship between Ultrasound of the Foot and Anthropometric Parameters

**Figure 2:**
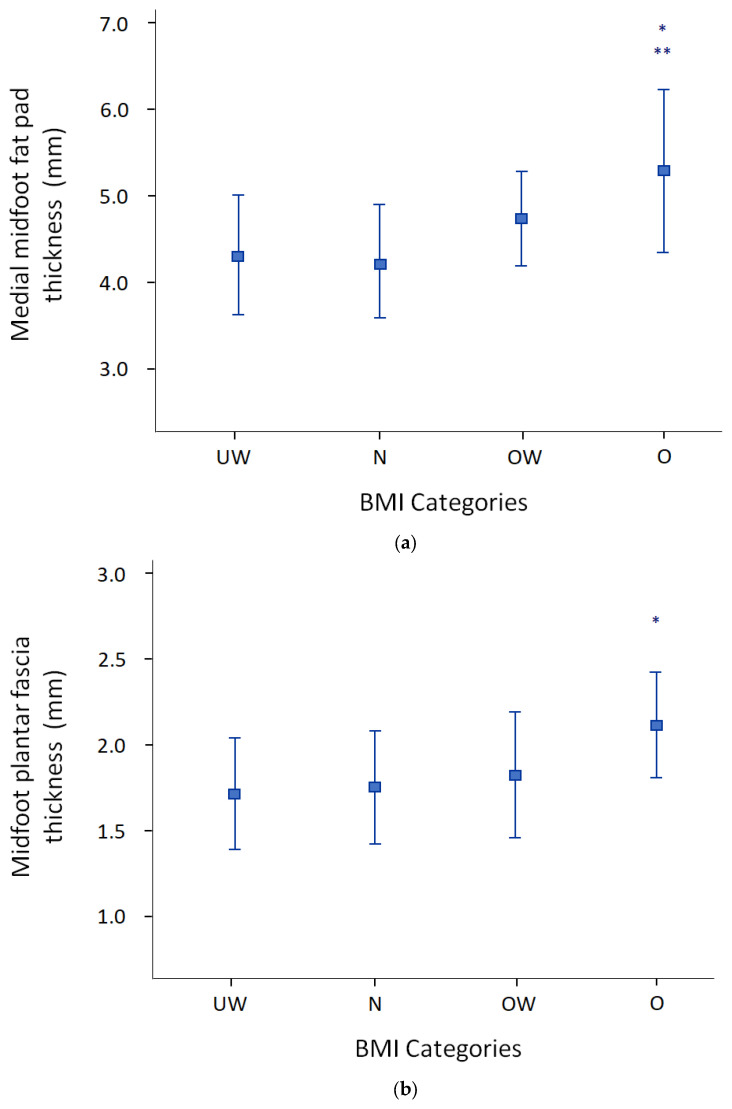
Ultrasonographical measurements MMFP (**a**) and MPF (**b**) of the foot stratified by BMI categories. UW, underweight; *n*, normal weight, OW, overweight; O, obese. * *p* < 0.05 vs. UW; ** *p* < 0.05 vs. N.

The TD1 children had similar MMFP thickness compared to the controls (4.80 ± 0.92 mm vs. 4.40 ± 0.64; *p* = 0.16). Similarly, there was no difference in MFP thickness between the two groups (1.90 ± 0.39 mm vs. 1.79 ± 0.31 mm; *p* = 0.4). Also, no correlation was found between HbA1c and either MMFP or MFP (data not shown). The MMFP thickness showed a moderate positive association with BMI (r = 0.66, *p* <0.001), WC (r = 0.68, *p* <0.001), and WHtR (r = 0.59, *p* < 0.001). The MPF thickness showed a low positive correlation with the MMFP thickness (r = 0.34, *p* = 0.027), BMI (r = 0.38, *p* = 0.013), WC (r = 0.38 *p* = 0.013), and WHtR (r = 0.34, *p* = 0.026). MMFP and MPF values progressively increased within the BMI categories (*p* = 0.02 and *p* = 0.04, respectively), with obese and overweight children showing higher values than normal weight and underweight children (Figure 2).

## 4. Discussion

In our cohort, the children with T1D had similar plantar tissue thickness (aponeurosis and adipose tissue) compared with those in the control group. Plantar tissue thickness increased proportionally to body mass so children with overweight and obesity had fatter feet and ticker plantar aponeurosis compared to their leaner peers.

To our knowledge, this is the first study that included both prepubertal children and adolescents (age range from 7 and 16 years) to determine, through clinical and ultrasound measurements, possible structural alterations in the growing foot of pediatric patients with T1D stratified by BMI. Previous studies have included either children up to 10 years [5] or adolescents only [11]. However, since the end of the growth period occurs around the age of 16, it seemed useful and necessary to study pediatric patients up to this age.

Our findings show an increase in plantar adipose tissue thickness in those children with elevated body mass compared to normal weight and underweight children. However, no association between anthropometric measures and static food posture, as measured by the FPI-6, was seen. These results support previous findings [5,6], which suggest that MMFP reflects adiposity rather than having a structural supportive function on the growing foot. In accordance with Riddiford-Harland et al. [6], we also found that the fat pad does not disappear during childhood with the development of MLA, as was hypothesized in previous studies [1]. In our cohort, the MMPF thickness range varied from 3.6 mm to 5.16 mm in children under 12 years and from 3.6 mm to 7.2 mm in adolescents.

To understand the functional and clinical relevance of the increased plantar fat tissue in overweight and obese subjects, we used ultrasound to study the MPF thickness. We found a positive correlation between the plantar fascia thickness, the plantar fat thickness, and anthropometric parameters. The plantar fascia (or aponeurosis) is a robust structure of the connective tissue that runs almost the entire length of the plantar surface of the foot and supports the plantar longitudinal arch [18]. Plantar fasciopathy is one of the most frequent diseases affecting plantar aponeurosis. Elevated BMI is recognized as a favoring factor of plantar fasciopathy [19,20], due to incremental pressures at the foot level generated by the excess body mass [5], and possibly due to an increased mechanical traction load within the aponeurosis [11]. In the case of a plantar fasciopathy, the ultrasound examination shows a thickening greater than 5 mm of the plantar fascia insertion, which loses the normally organized ligamentous architecture [18]. Griffith et al. [21] state that normal MPF appears as a sharply defined echogenic band of 1–2 mm thick, and the flexor brevis muscle of the toes adheres to the fascia’s deep surface. In our cohort, the MPF thickness range varied from 1.28 mm to 2.7 mm in children and from 1.08 mm to 2.6 mm in adolescents. As expected, the increase in anthropometric indices was associated with greater MPF thickness, a result more evident in obese patients than non-obese patients. Furthermore, as there was an increase in the MPF thickness, increasing values of MMFP thickness were recorded. Our findings and those reported by Riddiford-Harland et al. [5] would suggest that the thickening of the plantar aponeurosis is adaptive and consequent in part to the pressure exerted by the excess body mass weighing on the structures of the foot and in part to the pressure exerted by the plantar fat pad in the midfoot region. Although none of our patients reported pain in either the foot or the lower limb at the time of the evaluations, we cannot exclude that, if their excess body mass persists over time, they may suffer from pain, and injuries, and develop plantar fasciopathy. Also, the greater the excess adiposity and the longer the exposure to this excessive load, the greater the chance of developing plantar lesions [5].

According to our findings, the thickness of MPF in young people does not seem to be influenced by diabetic pathology but is correlated with BMI and MMFP. However, this result in our participants with diabetes might have been influenced by their good metabolic control (mean HbA1c 6.3%) and the short duration of the disease (5.7 years). Peripheral neuropathy is the main complication of diabetes and predisposes to lower extremity alterations [7]. The prevalence of neuropathy varies depending on the severity and duration of diabetes. In a recent cohort of children and adolescents with T1D, those with peripheral neuropathy had higher mean HbA1c levels (10.5% vs. 8.6%) and disease duration (11.3 vs. 7.4 years) compared to those without neuropathy [22].

Indeed, there is a study showing a greater plantar fascia thickness in adolescents with diabetes compared to healthy controls. Duffin et al. [11] have recruited 216 young people with diabetes (median disease duration 6 years; median lifetime HbA1c 8.6%) and 57 controls and have found that the plantar fascia was 0.1 mm thicker in adolescents with T1D than in controls without diabetes (*p* < 0.05). However, no difference in skin thickness, peak pressure, or pressure time integrals was found between the two groups, indicating that the thickening of the plantar aponeurosis did not alter the mechanical function of the foot. Of note, these researchers found a direct and significant association between the thickness of the aponeurosis and high BMI (i.e., one unit increase in BMI corresponded to a 0.011 mm increase in plantar aponeurosis thickening (*p* = 0.02)), suggesting that the thickening of the plantar fascia could also be consequent to an excess of body mass [11].

In this study, we compared for the first time the clinical and ultrasound measurements of the foot with the WHtR index, a simple anthropometric parameter used in children and adults to diagnose central obesity. Higher values of WHtR are predictors of obesity-related cardiovascular comorbidities. Although the BMI remains the method for determining overweight and obesity, data are suggesting the superiority of WHtR compared to BMI, since the former includes WC measured at navel height in its calculation [13]. In our cohort, WHtR is positively associated with lower arch height (measured by the NDT), thicker plantar adipose tissue, and thicker plantar aponeurosis. However, despite the correlation between WHtR and NDT, which would associate lower longitudinal arch height and pronated feet with central obesity, there is no significant correlation between WHtR and flatfeet as measured by FPI-6. In this study, the NDT and FPI-6 values are positively associated, regardless of BMI categories, which is in line with what is reported in the literature [16]. Therefore, we were unable to affirm that children and adolescents belonging to the overweight and obese categories have flatter feet compared to their leaner counterparts, as previously assumed [6]. These results, however, may have been influenced by the limited number of subjects and the characteristics of the sample examined. Although the FPI-6 was shown to have good reliability regardless of the clinical experience of the operator [23], it can be influenced by the morphology of the soft tissues, especially in overweight or obese subjects [24], and its use in children is less extensive than in adults [25]. Nevertheless, a recent analysis in a large cohort of 728 healthy children aged from 3 to 15 years, found no association between a flatter foot posture, as measured by FPI-6, and BMI [25].

This study has some limitations. First, the sample size is relatively small, due to the prospective recruitment of children with T1D in a relatively short time (i.e., three months). Second, our patients with T1D had relatively short duration of the disease and good metabolic control. Third, we did not include static and dynamic baropodometric analyses. Finally, a potential limitation comes from the study of the plantar fascia only in correspondence with the midfoot. However, we decided to focus on midfoot tissues since these are currently underrepresented in studies evaluating the impact of body mass on children’s foot posture and structures.

## 5. Conclusions

This study shows the usefulness of ultrasound in evaluating the foot of pediatric patients, being a non-invasive, simple, repeatable, and well-accepted method. Both prepubertal children and adolescents with T1D can have structural alterations of plantar soft tissues which seem directly related to the increase in body mass rather than diabetes pathology. Obesity is associated with fat feet and increased midfoot plantar fascia thickness, which can predispose to fasciopathy. This is particularly important in children with diabetes since they are naturally prone to foot injuries from minor trauma and neuropathic ulceration later in life. Ultrasound studies evaluating larger cohorts of children and adolescents with and without T1D should be performed to further investigate the effect of excess body fat on plantar soft tissue, possibly including static and dynamic baropodometric analyses, WHtR assessment, and long-term follow-up into adulthood.

## Figures and Tables

**Table 1:** Demographics, clinical and ultrasonographical characteristics of the study population.

Characteristics	Total (*n* = 43)	T1D (*n* = 29)	Controls (*n* = 14)	*p*-Value
Age (years)	12.9 (2.6)	12.86 (2.35)	12.93 (2.74)	0.931
Male, *n* (%)	20 (46.5)	16 (55)	7 (50)	0.75
Height (cm)	157.7 (15)	158 (15)	155 (16)	0.487
Weight (kg)	64.2 (27.6)	68.73 (29)	54.93 (22.64)	0.126
WC (cm)	86.4 (19.1)	89.74 (20.39)	79.36 (14.13)	0.094
WHtR (cm)	0.5 (0.1)	0.56 (0.11)	0.51 (0.06)	0.087
BMI (kg/m^2^)	24.9 (7.6)	26.48 (8.12)	21.76 (5.49)	0.057
Underweight, *n* (%)	11 (25.6)	5 (17)	6 (43)	0.24
Normal weight, *n* (%)	10 (23.3)	7 (24)	3 (21)	
Overweight, *n* (%)	9 (20.9)	6 (21)	3 (21)	
Obese, *n* (%)	13 (30.2)	11 (38)	2 (14)	
r-NDT (mm)	11.4 (3.4)	11.72 (3.62)	10.86 (2.77)	0.435
l-NDT (mm)	12.0 (2.9)	12.24 (3.16)	11.50 (2.35)	0.441
r-FPI6	6.9 (3.4)	7.24 (3.63)	6.14 (2.98)	0.332
l-FPI6	7.4 (3.5)	7.93 (3.61)	6.21 (2.94)	0.130
r-MMFP (mm)	4.8 (0.9)	4.90 (1.02)	4.52 (0.70)	0.222
l-MMFP (mm)	4.6 (0.9)	4.69 (0.93)	4.28 (0.76)	0.158
MMFP total (mm)	4.7 (0.9)	4.80 (0.92)	4.40 (0.64)	0.159
r-MPF (mm)	1.8 (0.4)	1.85 (0.41)	1.76 (0.32)	0.507
l-MPF (mm)	1.9 (0.4)	1.94 (0.44)	1.81 (0.38)	0.361
MPF total (mm)	1.9 (0.4)	1.90 (0.39)	1.79 (0.31)	0.400

BMI, Body mass index; l-, left foot; MMFP, Medial Midfoot Fat Pad; MPF, Midfoot Plantar Fascia; NDT, Navicular Drop Test; r-, right foot; T1D, type 1 diabetes; WC, waist circumference; WhtR, waist-to-heigh ratio (WHtR). Shown are *n* (%) for categorical variables and mean ± SD for continuous variables. Statistically significant differences (*p* < 0.05) between the two study groups are depicted in bold font.

**Table 2:** Pearson’s correlation analysis between clinical/demographic/anthropometric factors.

Factor	Statistics	Age	Weight	Height	BMI	WC	WHtR	r-NDT	l-NDT	r-FPI6	l-FPI6	MMFP Tot	r-MMFP	l-MMFP	MPF Tot	r-MPF	l-MPF
Age	Pearson’s r	1	0.562 **	0.865 **	0.297	0.362 *	−0.031	−0.178	−0.142	−0.138	−0.107	0.278	0.238	0.284	0.084	0.070	0.057
	*p*-value		<0.001	<0.001	0.053	0.017	0.842	0.252	0.363	0.379	0.493	0.071	0.125	0.065	0.591	0.665	0.718
Weight	Pearson’s r	0.562 **	1	0.743 **	0.928 **	0.929 **	0.704 **	0.144	0.143	0.098	0.073	0.668 **	0.608 **	0.643 **	0.357 *	0.376 *	0.278
	*p*-value	<0.001		<0.001	<0.001	<0.001	<0.001	0.356	0.360	0.530	0.642	<0.001	<0.001	<0.001	0.019	0.015	0.075
Height	Pearson’s r	0.865 **	0.743 **	1	0.465 **	0.541 **	0.122	−0.132	−0.067	−0.124	−0.059	0.383 *	0.313 *	0.406 **	0.197	0.225	0.121
	*p*-value	<0.001	<0.001		0.002	<0.001	0.435	0.397	0.670	0.428	0.707	0.011	0.041	0.007	0.205	0.157	0.444
BMI	Pearson’s r	0.297	0.928 **	0.465 **	1	0.953 **	0.883 **	0.258	0.255	0.245	0.164	0.659 **	0.629 **	0.603 **	0.377 *	0.376 *	0.316 *
	*p*-value	0.053	<0.001	0.002		<0.001	<0.001	0.094	0.099	0.113	0.293	<0.001	<0.001	<0.001	0.013	0.016	0.042
WC	Pearson’s r	0.362 *	0.929 **	0.541 **	0.953 **	1	0.898 **	0.225	0.304 *	0.145	0.183	0.681 **	0.665 **	0.606 **	0.376 *	0.390 *	0.300
	*p*-value	0.017	<0.001	<0.001	<0.001		<0.001	0.148	0.048	0.352	0.240	<0.001	<0.001	<0.001	0.013	0.012	0.054
WHtR	Pearson’s r	−0.031	0.704 **	0.122	0.883 **	0.898 **	1	0.317 *	0.392 **	0.248	0.258	0.587 **	0.610 **	0.484 **	0.339 *	0.337 *	0.288
	*p*-value	0.842	<0.001	0.435	<0.001	<0.001		0.038	0.009	0.109	0.094	<0.001	<0.001	0.001	0.026	0.031	0.064
r-NDT	Pearson’s r	−0.178	0.144	−0.132	0.258	0.225	0.317 *	1	0.805 **	0.606 **	0.467 **	0.179	0.023	0.319 *	0.175	0.211	0.115
	*p*-value	0.252	0.356	0.397	0.094	0.148	0.038		<0.001	<0.001	0.002	0.251	0.882	0.037	0.262	0.186	0.469
l-NDT	Pearson’s r	−0.142	0.143	−0.067	0.255	0.304 *	0.392 **	0.805 **	1	0.657 **	0.726 **	0.243	0.142	0.318 *	0.165	0.213	0.087
	*p*-value	0.363	0.360	0.670	0.099	0.048	0.009	<0.001		<0.001	<0.001	0.116	0.363	0.038	0.291	0.180	0.585
r-FPI6	Pearson’s r	−0.138	0.098	−0.124	0.245	0.145	0.248	0.606 **	0.657 **	1	0.754 **	0.116	0.008	0.216	0.161	0.233	0.082
	*p*-value	0.379	0.530	0.428	0.113	0.352	0.109	<0.001	<0.001		<0.001	0.457	0.962	0.165	0.303	0.143	0.605
l-FPI6	Pearson’s r	−0.107	0.073	−0.059	0.164	0.183	0.258	0.467 **	0.726 **	0.754 **	1	0.220	0.164	0.250	0.120	0.158	0.063
	*p*-value	0.493	0.642	0.707	0.293	0.240	0.094	0.002	<0.001	<0.001		0.156	0.294	0.106	0.445	0.324	0.690
MMFP tot	Pearson’s r	0.278	0.668 **	0.383 *	0.659 **	0.681 **	0.587 **	0.179	0.243	0.116	0.220	1	0.939 **	0.931 **	0.337 *	0.416 **	0.214
	*p*-value	0.071	<0.001	0.011	<0.001	<0.001	<0.001	0.251	0.116	0.457	0.156		<0.001	<0.001	0.027	0.007	0.173
r-MMFP	Pearson’s r	0.238	0.608 **	0.313 *	0.629 **	0.665 **	0.610 **	0.023	0.142	0.008	0.164	0.939 **	1	0.748 **	0.356 *	0.396 *	0.264
	*p*-value	0.125	<0.001	0.041	<0.001	<0.001	<0.001	0.882	0.363	0.962	0.294	<0.001		<0.001	0.019	0.010	0.091
l-MMFP	Pearson’s r	0.284	0.643 **	0.406 **	0.603 **	0.606 **	0.484 **	0.319 *	0.318 *	0.216	0.250	0.931 **	0.748 **	1	0.271	0.371 *	0.132
	*p*-value	0.065	<0.001	0.007	<0.001	<0.001	0.001	0.037	0.038	0.165	0.106	<0.001	<0.001		0.079	0.017	0.404
MPF tot	Pearson’s r	0.084	0.357 *	0.197	0.377 *	0.376 *	0.339 *	0.175	0.165	0.161	0.120	0.337 *	0.356 *	0.271	1	0.922 **	0.936 **
	*p*-value	0.591	0.019	0.205	0.013	0.013	0.026	0.262	0.291	0.303	0.445	0.027	0.019	0.079		<0.001	<0.001
r-MPF	Pearson’s r	0.070	0.376 *	0.225	0.376 *	0.390 *	0.337 *	0.211	0.213	0.233	0.158	0.416 **	0.396 *	0.371 *	0.922 **	1	0.727 **
	*p*-value	0.665	0.015	0.157	0.016	0.012	0.031	0.186	0.180	0.143	0.324	0.007	0.010	0.017	<0.001		<0.001
l-MPF	Pearson’s r	0.057	0.278	0.121	0.316 *	0.300	0.288	0.115	0.087	0.082	0.063	0.214	0.264	0.132	0.936 **	0.727 **	1
	*p*-value	0.718	0.075	0.444	0.042	0.054	0.064	0.469	0.585	0.605	0.690	0.173	0.091	0.404	<0.001	<0.001	

* Significance < 0.05 (two tailed); ** Significance < 0.01 (two tailed).

## Data Availability

The data that support the findings of this study are available on request from the corresponding author [P.C.].

## References

[1] Riddiford-Harland D.L., Steele J.R., Storlien L.H. (2000). Does obesity influence foot structure in prepubescent children?. Int. J. Obes. Relat. Metab. Disord..

[2] McCarthy H.D. (2014). Measuring growth and obesity across childhood and adolescence. Proc. Nutr. Soc..

[3] Ke D., Lu D., Cai G., Zhang J., Wang X., Suzuki K. (2020). Accelerated skeletal maturation is associated with overweight and obesity as early as preschool age: A cross-sectional study. BMC Pediatr..

[4] Dowling A.M., Steele J.R., Baur L.A. (2001). Does obesity influence foot structure and plantar pressure patterns in prepubescent children?. Int. J. Obes. Relat. Metab. Disord..

[5] Riddiford-Harland D.L., Steele J.R., Baur L.A. (2011). Medial midfoot fat pad thickness and plantar pressures: Are these related in children?. Int. J. Pediatr. Obes..

[6] Riddiford-Harland D.L., Steele J.R., Baur L.A. (2011). Are the feet of obese children fat or flat? Revisiting the debate. Int. J. Obes..

[7] Tuttolomondo A., Maida C., Pinto A. (2015). Diabetic foot syndrome: Immune-inflammatory features as possible cardiovascular markers in diabetes. World J. Orthop..

[8] Tüzün B., Tüzün Y., Dinççağ N., Minareci O., Oztürk S., Yilmaz M.T., Satman I., Yazici H. (1995). Diabetic sclerodactyly. Diabetes Res. Clin. Pract..

[9] Abouaesha F., van Schie C., Griffiths G., Young R., Boulton A. (2001). Plantar tissue thickness is related to peak plantar pressure in the high-risk diabetic foot. Diabetes Care.

[10] Rosenbloom A., Silverstein J., Lezotte D., Richardson K., McCallum M. (1981). Limited joint mobility indicates increased risk of microvascular disease. N. Engl. J. Med..

[11] Duffin A.C., Lam A., Kidd R., Chan F.K.A., Donaghue K.C. (2002). Ultrasonography of plantar soft tissues thickness in young people with diabetes. Diabet. Med..

[12] Cacciari E., Milani S., Balsamo A., Spada E., Bona G., Cavallo L., Cerutti F., Gargantini L., Greggio N., Tonini G. (2006). Italian cross-sectional growth charts for height, weight and BMI (2 to 20 yr). J. Endocrinol. Investig..

[13] Nambiar S., Truby H., Abbott R.A., Davies P.S.W. (2009). Validating the waist-height ratio and developing centiles for use amongst children and adolescents. Acta Paediatr..

[14] Morrison S.C., Ferrari J. (2009). Inter-rater reliability of the Foot Posture Index (FPI-6) in the assessment of the paediatric foot. J. Foot Ankle Res..

[15] Brody D.M. (1982). Techniques in the evaluation and treatment of the injured runner. Orthop. Clin. N. Am..

[16] Zuil-Escobar J.C., Martinez-Cepa C.B., Martin-Urrialde J.A. (2019). Evaluating the Medial Longitudinal Arch of the Foot: Correlations, Reliability, and Accuracy in People with a Low Arch. Phys. Ther..

[17] Riddiford-Harland D.L., Steele J.R., Baur L.A. (2007). The use of ultrasound imaging to measure midfoot plantar fat pad thickness in children. J. Orthop. Sports Phys. Ther..

[18] McNally E.G., Shetty S. (2010). Plantar fascia: Imaging diagnosis and guided treatment. Semin. Musculoskelet. Radiol..

[19] Griffith J.F., Wong T.Y., Wong S.M., Wong M.W., Metreweli C. (2002). Sonography of plantar fibromatosis. AJR Am. J. Roentgenol..

[20] Boules M., Batayyah E., Froylich D., Zelisko A., O’Rourke C., Brethauer S., El-Hayek K., Boike A., Strong A.T., Kroh M. (2018). Effect of Surgical Weight Loss on Plantar Fasciitis and Health-Care Use. J. Am. Podiatr. Med. Assoc..

[21] Taş S., Bek N., Ruhi Onur M., Korkusuz F. (2017). Effects of Body Mass Index on Mechanical Properties of the Plantar Fascia and Heel Pad in Asymptomatic Participants. Foot Ankle Int..

[22] Ghaemi N., Hasanabadi H., Ashrafzadeh F., Sarvari S., Rahimi H., Hashemian S. (2018). Peripheral Neuropathy in Children and Adolescents with Insulin-dependent Diabetes Mellitus. Iran. J. Child Neurol..

[23] Terada M., Wittwer A.M., Gribble P.A. (2014). Intra-rater and inter-rater reliability of the five image-based criteria of the foot posture index-6. Int. J. Sports Phys. Ther..

[24] Hegazy F.A., Aboelnasr E.A., Salem Y., Zaghloul A.A. (2020). Validity and diagnostic accuracy of foot posture Index-6 using radiographic findings as the gold standard to determine paediatric flexible flatfoot between ages of 6–18 years: A cross-sectional study. Musculoskelet. Sci. Pract..

[25] Evans A.M., Karimi L. (2015). The relationship between paediatric foot posture and body mass index: Do heavier children really have flatter feet?. J. Foot Ankle Res.

